# The Human Eye

**Published:** 1879-03

**Authors:** H. F. Scott

**Affiliations:** Atlanta, Ga.


ATLANTA

Medical and Surgical Journal.

Vol. XVI.] MARCH—1879. [No. 12

### Original Communications.

### THE HUMAN EYE.

By H. F. SCOTT, M.D., Atlanta, Ga.

*Read before the Academy of Medicine.*

Having been appointed by President Todd, to read an Essay before the Academy, you will pardon my selecting a subject appertaining to my speciality of diseases of the eye.

You will doubtless agree with me, when I say that the grandest of all optical instruments is the human eye.

The telescope, which sweeps the sidercal heavens with a glance, and resolves far distant nebulæ in the twinkling of an eye, is a marvelous product of human ingenuity.

The microscope, which magnifies a razor's edge to a finger's breadth, and reveals the minutest organisms of animal and vegetable life, is scarcely less wonderful; but the human eye exhibits a higher mechanism than either or both of these notable contrivances of scientific skill.

In discussing briefly the eye, its use and abuse, you will pardon us for prefacing our remarks with a bare outline of the anatomy and physiology of this most important organ.

The eye is a globe, spheroidal in shape, consisting of the sclerotic and choroid coats, of the cornea, which in construction closely resembles a watch crystal, and of the iris, a curious network of muscular fibres, interlaced with blood-vessels and nervous filaments, perforated by a pupil through which light is admitted into the inner chamber of the eye.

In addition, it has tbe aqueous humor, crystalline lens and the vitreous body, which serve as so many refracting media, and besides impart shape and consistency to the eye.

The eye, as thus constituted, is essentially a photographic *camera-obscura.*

In the rear, however, of the chamber of the eye, we meet with that remarkable expansion of the optic nerve, called the retina; its office is to receive the impressions of external objects, and by means of its conducting apparatus, to transmit them to the sensorium where they become objects of perception.

We are greatly indebted to Schulze for our present knowledge of the retina.

According to his analysis, it is composed of ten distinct layers, all contributing in a greater or lesser measure to the purposes of vision.

It remains to be stated that the eye is sustained and operated by six muscles, the four recti-muscles—superior, inferior, internal and external, and the two oblique—the superior and inferior.

It need hardly be said, that it is lodged in well-cushioned sockets, and yet further protected from injury by bony processes, by the eyelids and by glands whose secretions lubricate and cleanse the eye.

For the purposes of distinct vision, the crystalline lens is of transcendant importance.

By its refracting power it forms a sharp, well defined image of the external object upon the retina, when otherwise the image would be blurred and greatly confused.

The structure of the lens is not homogeneous, but it decreases in density from the centre to the circumference.

In the living eye it is perfectly pellucid, but shortly after death, or when subjected to the action of heat or alcohol, it becomes opaque and impervious to the rays of light.

Upon this lens, also, depends the power of accommodation; when an object is brought near the eye, its convexity is increased by the action of the fibres of the ciliary muscle, and the object is clearly perceived at a short distance.

This faculty of accommodation or adjustment is marvelous beyond expression. With equal facility it catches the light of Sirius several billions of miles from the earth, and perceives with distinctness the form and color of the violet at a distance of six inches.

Without wearying you further with dry anatomical details, suffer us to speak at greater length of the uses of the eye. Much has been sung and written of the triumphs of blindness. The world is familiar with the story of the blind old bard of Scio's rocky isle, and of him not less gifted who sung of man's first disobedience and the fruit of that forbidden tree whose mortal taste brought death into the world with all our woe.

Most of you have heard of Sanderson, the blind mathematician, and of Huber, the Swiss naturalist, who taught us the husbandry of bees.

Not a few have read of Nydia, the blind flower-girl of Pompeii, who guided Glaucus, the Athenian, in perfect safety through the storm of fire, ashes and scoriæ with which Vesuvius entombed the queenly city for a thousand years; and of that greatest prodigy, Laura Brigman, who, though blind and deaf, acquired a competent knowledge of history, geography and arithmetic by the tips of her fingers.

We see in these instances an illustration of that law of compensation by which a merciful Providence very nearly equalizes the varied lots and conditions of men.

Let us not too hastily conclude that the eye is of secondary importance—after all, it is the chief inlet of knowledge of the external world; in this respect it far exceeds in value all the other senses.

If the human skull be indeed the dome of thought and palace of the soul, much more is the eye the window through which the mind itself looks out upon the material universe and becomes cognizant of its beauty and its glory—differing in its shade through all the gamut of colors, from the deep blue eye of an Annie Laurie to the languishing dark eye of an Andalusian maid, which is beyond all else the truest exponent of the character.

No one who has read Milton's exquisite sonnet on his own blindness, or his yet sublimer apostrophe to Light in the earlier cantos of "Paradise Lost" can fail to perceive that if the eye is once destroyed, creation, as to form, coloring and the thousand other charms, becomes a universal blank.

It may be well to say, in speaking of the uses of the eye, that we are morally responsible, in a measure, for what we see or fail to see—there is more in our subjective states than we often dream of.

Shakespeare truthfully says that the lover sees Helen's beauty in a brow of Egypt, and you all remember that Titania deemed Bottom a paragon of beauty, despite his assenine ears.

Very much of our success in business or professional pursuits will depend upon the use we make of our eyes. Many things are best learned from observation. Quick perception, associated with a habit of close, unfaltering attention, is the great secret of genius itself. Parents therefore owe it to themselves, but far more to their children, to care for the eyes. Hundreds of dollars, however, are expended on the teeth, to where one dollar is expended in securing the professional services of an oculist. Much of this neglect is due to popular ignorance, but is none the less damaging to the child.

Some eye diseases are remarkably insidious in their approaches, and if not promptly arrested, in a very little while they become incurable. Prominent among these is Glaucoma. In its acute form there is high inflammatory action, attended with severe pain; yet, in these cases even, an expectant treatment is often adopted which merely soothes and palliates, or, at the most, postpones the inevitable result.

In the chronic form of glaucoma the danger is seldom appreciated, and this fact, coupled with the dread of a surgical operation, occasions delay that is not less fatal to the vision of the affected eye. A timely resort to the simple and almost uniformly successful operation of Iridectomy, will at least preserve what vision may have survived the ravages of the disease.

You will pardon the seeming egotism of the remark when I say that I myself have witnessed numbers of such operations by Arlt at his clinics in Vienna, and in but few instances was there a failure, and then but a partial failure. In all cases everything depends on the prompt application of the knife. A very high authority, indeed, says that in estimating available time in glaucoma, hours must be reckoned as days, and in the fulminating form, minutes must be measured as hours.

Another disease, which nearly always develops itself in childhood, is less insidious and less hurtful than glaucoma, but yet demands the immediate attention of the professional oculist. We refer to Strabismus, or squint, both convergent and divergent. In not a few cases the disease is the result of local paralysis, and in some of these cases, the usual severance of the muscle supposed to be at fault is a grave mistake; it inflicts needless suffering without producing parallelism.

In such, a constitutional treatment is indicated and is very often beneficial, yet, in the majority of instances the atropine treatment, accompanied by the use of suitable glasses, will be the most reliable remedy. Of course we recognize the necessity for the knife in the larger number of cases of strabismus. Our special object, however, in this connection, is to insist that in every case a skilled oculist be consulted before the vicious habit entails changes which are beyond the hope of remedy.

Another form of eye disease demands prompt attention. We allude to Sympathetic Ophthalmia. Any injury done to one eye, especially if it involves the ciliary region, will be liable to develop sympathetic inflammation of a peculiarly obstinate and destructive character in the uninjured eye. In such an event, the *immediate enucleation of the injured eye* is imperatively called for. This affection sometimes follows an operation for the extraction of cataract; but it holds likewise in regard to all mechanical injuries.

In these cases (and they are constantly occurring) the patient should seek the counsel of an oculist, and he, if an expert, will extirpate, when needful, the offending organ and so prevent a total loss of vision.

If deemed important for the purposes of this essay, I might speak in detail of Pterygium, conjunctivitis, cataract, etc., which are well known and comparatively well understood. Their presence is readily detected, and the needed treatment is seldom so urgent as in the forms of disease just mentioned.

The remark which we made at the outset, that the eye was the grandest of all optical instruments, was strictly true in the sense there intended, but in another sense it needs material qualification.

Statistics show that there is only about one person in every thousand that needs the restraint and regimen of a lunatic asylum, but deQuincey was not wide of the mark when he said that not more than one person in sixty is perfectly sane.

So, while there are comparatively few who require the professional services of an oculist, yet there is only a small percentage of our population whose vision is perfect. A large majority of those who compose this or any similar assemblage are really laboring under some infirmity of vision.

The ideal eye, technically called emmetropic, is one of the rarest bestowments of the Great Creator; much the larger portion have either myopic or hypermetropic eyes—in popular parlance, they are either far-sighted or short-sighted.

The deviation from the normal standard may be but slight, and the individual may remain unconscious of the defect for years, but it nevertheless exists, and is the source of more or less discomfort and incapacity.

The ophthalmoscope in the hands of a skillful oculist very often reveals defects of this sort that had not been previously suspected.

In many cases these organic defects are congenital and in other cases they are the outcome of long-continued functional disturbances. In all cases, however, they may be benefited, if not fully remedied, by proper treatment or the use of suitable glasses.

It will not be out of place here to notice a few of the causes of those diseases of the eye which are not strictly congenital. One of the most frequent of these causes, as will readily occur to all of you, is habitually over-taxing the eye. The eye, like every other organ, needs intervals of rest; it is not more certain that Assyrian or Egyptian sun will develop ophthalmia of a malignant type, than it is that reading by an insufficient light, whether natural or artificial, or excessive reading by an adequate light, congests the organs of vision.

The old maxim, *ubi irritus ibi fluxus*, applies to this as well as to every other inflammatory action.

A pleasant thing, says an inspired writer, it is for the eye to see the sun, but the softest sunlight of a May morning is stimulating to the optic nerve, and if too long continued, will irritate and inflame its delicate tissues. This injury is vastly intensified by the atmosphere of a crowded hall, where "bright lamps shine o'er fair women and brave men, and soft eyes speak love to eyes that speak again."

We would lay no needless restraint upon the innocent and healthful recreation of the young, but even a blind man can see that midnight revelry in a poisoned atmosphere, amidst the flashing of wine cups and the glare of gaslight, is doing no little to produce premature blindness and even premature death. The Norman Curfew, hated as it was by our Saxon ancestors, would in these respects be a blessing to this age and country.

Another grave abuse of the eye is the habitual reading of books and magazines printed in an exceedingly small type; some of the cheap editions of our favorite poets and novelists are both a typographical swindle and a medical abomination; if they had been studiously contrived to bring about one or another of the diseases known to the professional oculist, they could not have been more successful.

Our sanitary boards, who are devising ways and means to stay the march of pestilence, ought to bestow at least a passing notice upon this widely prevalent evil. And those who have in charge our public libraries should discountenance these publications, which, under the pretext of cheap literature, are sowing broadcast the seeds of eye disease amongst the youth of both sexes.

It may be stated in general terms, that any influence which impairs the bodily health, thereby lowering the vitality of the system, is damaging in a greater or less degree to the eye.

In harmony with this proposition is the fact that various forms of eye disease become almost epidemical in our crowdsd school-rooms and college halls. Other causes probably increase this tendency, yet much of it may be fairly attributed to imperfect ventilation and indigestion, caused by the sedentary habits of the pupils.

Our boards of education, and all who have charge of our public and private schools, need to be especially watchful against all these sources of injury to the delicate tissues of the eye.

We cannot too earnestly admonish all against any sort of reliance upon the various nostrums that are placarded at street corners and announced through the columns of the newspapers. In every case of eye disease, the patient should seek the counsel of a competent physician—a professional oculist if one is within reach. This advice, if followed, will save time and money, and perhaps the eye.

Avoid itinerant opticians, who peddle Scotch-pebble glasses, and who have no knowledge of the eye and no understanding of its needs. It requires as much professional skill to rightly adjust glasses to the eye as to remove a cataract. Badly adjusted, glasses are positively harmful, while well-adjusted, they are helpful to imperfect vision, and contribute greatly to the patient's comfort and capacity for business.

In speaking, a little while ago, of the treatment of certain forms of strabismus, I alluded to the *atropine treatment*, and in bringing these remarks to a close, I can not forbear saying more of this, the most important remedy in the armamentarium of the oculist, without the knowledge of which we would be like a ship at sea without a compass.

In the form of a solution of the *neutral* sulphate of the strength of from ½ a grain to 4 grains to an ounce of distilled water, it is applicable in almost every affection of the eyeball, those alone in which there is a preternatural dilatation of the pupil being excepted. This remedy should be known and appreciated by every physician, for by its use we are enabled to paralyze for a time both the sphincter of the pupil and the ciliary muscle, thus placing the eye in a state of absolute repose.

The science of ophthalmology is still in its infancy. It has been said, indeed, that professional oculists were known amongst the Greeks and Romans, and that even the Egyptians were familiar with the leading principles of ophthalmology. How much of this is simply conjectural, if not truly fanciful, I shall not take time to discuss. Certain it is, that if the ancients possessed half the knowledge accredited to them, then ophthalmology deserves to rank amongst the lost arts of antiquity; for it is matter of history that during the 16th and 17th centuries and earlier half of the 18th century, the art was mainly in the hands of Quacks and Sciolists.

So general was the lack of information, that even the illustrious Boerhaave gravely affirmed that mercury would dissolve a cataract. To borrow his own Latin phraseology, *Mercuris saepe perfectus cataractas solvit.* Joseph Barth, a native of Malta, and afterwards a professor at Vienna, was the earliest scientific oculist of modern times. He was to ophthalmology what Lavoisier was to chemistry.

He popularized it and prepared the way for the advent of the von Græfes and Arlts, whose greater discoveries and achievements have been such signal blessings to the world.

At an early period of the present century a well-organized Eye Infirmary was established in London, which has done a great deal to disseminate light through all parts of the United Kingdom.

It is almost superfluous to say, however, that Germany has outstripped every other nation in this interesting field of investigation.

Much of this scientific advancement is due to the invention of the ophthalmoscope, which, as improved by

Loring, not only enables us to search the innermost recesses—the very adytum—of the chamber of the eyes and to know its condition as perfectly as the outer eye, but also to measure the degrees of refraction, so as to supplement the eye by artificial helps.

What we most need now for continued progress is a wholesome public sentiment which will appreciate true science and discountenance mercenary pretenders, whose treatment is as absurd as the conjurations of an Indian doctor or the prescriptions of a clairvoyant, but unfortunately not half so harmless.

It is a matter of sincere congratulation that so much has and may be done to alleviate suffering, and even heal deeply-seated diseases of the eye. We look, however, to yet nobler triumphs of professional skill in this department of science.

True the age of miracles is past, and we may not, therefore, hope to rival the Great Physician who could say to the blind, Bartimeus receive thy sight, and straightway it was done; or who, on another occasion, said to the nameless sufferer, who had been born blind, go wash in the pool of Siloam, and he went and washed, and came seeing.

We may, however, do much to soften the lot of that numerous class, who having eyes, see not or see dimly, and this labor, though inadequately remunerated, will make us like Him whose life-work was the doing of good, and thus, may we too, best fulfil the grander destinies of life, and thus discharge in some measure the debt we owe to our profession and to our common humanity.

